# A Streamlined High-Throughput Plasma Proteomics Platform for Clinical Proteomics with Improved Proteome Coverage, Reproducibility, and Robustness

**DOI:** 10.1021/jasms.3c00022

**Published:** 2023-03-28

**Authors:** Jongmin Woo, Qibin Zhang

**Affiliations:** † Center for Translational Biomedical Research, University of North Carolina at Greensboro, North Carolina Research Campus, Kannapolis, North Carolina 28081, United States; ‡ Department of Chemistry & Biochemistry, University of North Carolina at Greensboro, Greensboro, North Carolina 27402, United States

**Keywords:** human plasma proteome, DIA, high throughput, clinical proteomics

## Abstract

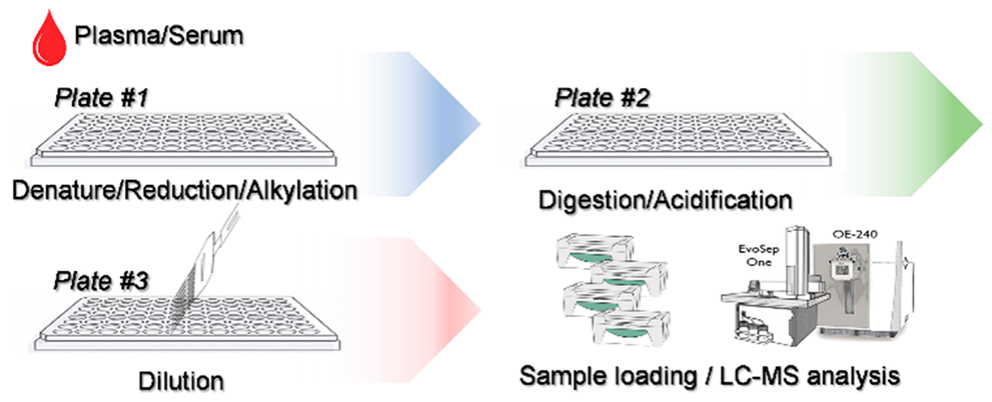

Mass spectrometry-based clinical proteomics requires high throughput, reproducibility, robustness, and comprehensive coverage to serve the needs of clinical diagnosis, prognosis, and personalized medicine. Oftentimes these requirements are contradictory to each other. We report the development of a streamlined High-Throughput Plasma Proteomics (sHTPP) platform for untargeted profiling of the blood plasma proteome, which includes 96-well plates and simplified procedures for sample preparation, disposable trap column for peptide loading, robust liquid chromatographic system for separation, data-independent acquisition in tandem mass spectrometry, and DIA-NN, FragPipe, and in-house peptide spectral library-based data analysis. Using the optimized platform at a throughput of 60 samples per day, over 600 protein groups including 57 FDA-approved biomarkers can be consistently identified from whole human plasma, and more than 85% of the detected proteins have 100% completeness in quantitative values across 300 samples. The balance achieved between proteome coverage, throughput, and reproducibility of this sHTPP platform makes it promising in clinical settings, where a large number of samples are to be measured quickly and reliably to support various needs of clinical medicine.

## 1 Introduction

Blood is the most common type of bio specimen for disease diagnosis, prognosis, and therapeutic monitoring. Besides various cell types, the vast constituents of blood include proteins, metabolites, and electrolytes that reflect the molecular profiles of the human physiology and pathology. Many mass spectrometry based assays have been developed to monitor the metabolites, such as in newborn metabolic screening.^1,2^ Although over one hundred proteins or enzymes were approved by the Food and Drug Administration (FDA) as biomarkers for various diseases,^3^ they are mainly determined by enzymatic or immunoassays in a targeted fashion. Application of LC–MS in measurement of proteins in a clinical setting is rare, despite the fact that proteins can be analyzed reliably using LC–MS.^4^ Compared to the immunoassays against a single target protein, mass-spectrometry based untargeted proteomics can quantify the changes of the proteome simultaneously, enables generating hypotheses about disease mechanism, and is a corner stone of the precision or personalized medicine.^5^

Using blood samples, particularly plasma or serum for more sensitive protein biomarker discovery, has been an intensive research area in mass spectrometry-based proteomics in the last two decades. Because disease-specific proteins in the early stage of the disease are in low abundance,^6^ to achieve higher protein coverage, plasma samples typically need to be depleted to remove the highly abundant proteins, and various depletion strategies were developed and applied for in-depth blood proteome coverage in discovery studies.^7−10^ While depletion is essential and has its merits to broaden proteome coverage, it is also acknowledged that lengthy sample preparation and expensive reagents are needed in the depletion process, and more importantly some proteins could be co-depleted with the abundant ones and accuracy in protein quantitation is compromised.^11,12^ As a result, measurement of naïve or non-depleted plasma gains popularity in clinical analysis, the so-called “rectangular” plasma proteome profiling strategy,^5^ where the protein marker discovery and validation are performed directly on large cohorts, is showing promise in translating laboratory discovery to clinical decision. This is especially critical in a pandemic where classifiers are urgently needed at the point-of-care to guide treatment decisions.^13,14^

Various LC–MS-based proteomics platforms have been developed to analyze the proteome in nondepleted plasma samples, identifying hundreds of proteins with good coverage of the FDA approved protein biomarkers.^13,15−20^ Traditionally, data-dependent acquisition (DDA) has been the standard sampling method in proteomics; however, its inherent issues in under sampling/missing values and in quantitation accuracy limit its application to highly quantitative clinical settings.^21^ Data-independent acquisition (DIA), which divides coeluted peptides into a series of overlapping mass windows before surveying all theoretical fragment ion spectra, was developed to overcome these issues, but successful implementation of DIA requires a comprehensive spectral library to interpret the more complex spectra acquired.^22−29^ In addition, various proteomic sample preparation procedures were developed to process plasma/serum. The complexity of sample preparation procedures can introduce variations and errors between experiment batches.^30^ Although batch effect normalization or correction may partially address these issues, it is always preferred to not manipulate the data after measurement.^31^ Fully automated robotic systems can alleviate the intensive labor and some of the human errors in sample preparation, but they may not be accessible to most of the laboratories.

LC–MS-based platform for high-throughput clinical proteomics should strike a balance between sensitivity, reproducibility, and robustness. Here we report the development of a streamlined high-throughput plasma proteomics (sHTPP) platform, which incorporates a simplified sample processing workflow using 96 well plates and easily accessible laboratory equipment, disposable trap column-based LC-DIA-MS/MS, and DIA-NN based data processing. The sHTPP platform was carefully evaluated, optimized, and validated using hundreds of plasma samples from a clinical study, demonstrating improved efficiency in sample preparation, increased depth in proteome coverage, and improved reproducibility and precision in protein identification and quantification.

## 2 Methods

### 2.1 Human Plasma/Serum Samples

Plasma and serum of healthy individuals were purchased from BioIVT (Westbury, NY). Low-oxylipin (LO) and high-oxylipin (HO) plasma samples were prepared by pooling plasma from previous studies.^32,33^ For method validation, 300 blood samples (Clinical trial #: NCT05407701) were collected longitudinally from 25 individuals using K2EDTA tubes and centrifuged at 1,500*g* for 20 min, and plasma was then collected and stored at −80 °C until analysis. For generating peptide spectral library, a pooled plasma sample was depleted to remove the top 14 most abundant proteins using a human 14 multiple affinity removal column (catalog no. 5188-6557, Agilent) according to the manufacturer’s instructions. Before proteomic processing, plasma/serum samples were centrifuged at 10,000*g* for 10 min to remove particles.

### 2.2 Proteomics Sample Preparation

#### 2.2.1 Microcentrifuge Tube-Based Sample Processing

Plasma or serum was placed in a 1.5 mL Protein LoBind microcentrifuge tube (catalog no. 00300108442, Eppendorf) containing 8 M urea and 10 mM dithiothreitol (DTT) in 50 mM triethylammonium bicarbonate (TEABC) and denatured/reduced for 1 h at 30 °C. The proteins were alkylated in 20 mM iodoacetamide (IAA) for 1 h at room temperature in the dark, followed by addition of 50 mM TEABC to dilute the urea concentration to less than 1 M, and a 1:10 enzyme-to-protein ratio of trypsin and lysin C mixture (catalog no. A41009, Thermo Scientific) was added. After incubation for 16 h, peptides were acidified in 1% formic acid (FA) before cleaning up with ISOLUTE C18 solid phase extraction column (catalog no. 220–0010-A, Biotage) for comparison of desalting-on and -off or stored at −20 °C until further processing.

#### 2.2.2 96-Well Plate-Based Sample Processing

**Figure 1:**
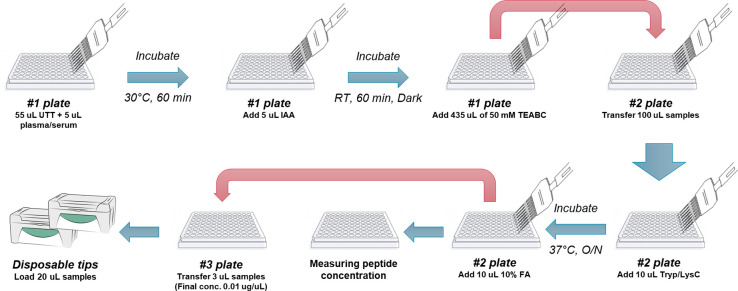
Illustration of the sample preparation steps in the sHTPP workflow.

The workflow for high-throughput proteomics sample preparation was optimized using 96-well plates to minimize processing steps and reduce the intra and interplate variations (Figure 1). An 8-channel pipet was used for most of the liquid handling except addition of individual samples. Briefly, 5 μL of plasma or serum was added to the wells of a 1 mL 96-well plate (catalog no. 260252, Thermo Scientific) containing 55 μL of 8 M urea and 10 mM DTT in 50 mM TEABC. Denaturation and reduction of the proteins were performed by incubation at 30 °C for 1 h, followed by alkylation in 20 mM IAA at room temperature for 1 h in dark. After diluting to below 1 M urea with 435 μL of 50 mM TEABC, 100 μL of each sample was transferred to a 200 μL 96-well plate (catalog no. 03-251-447, Thermo Scientific), then a mixture of trypsin/lysin C (1:10 enzyme-to-protein ratio) was added, and the plate was sealed with tape (catalog no. 03-252-444, Thermo Scientific) to prevent sample evaporation before overnight incubation at 37 °C in a microplate incubator (Incu-Mixer MP, Benchmark, Dawsonville, GA). Afterward, samples were acidified with 1% FA, and peptide concentrations were determined using BCA assay. To dilute each sample to 0.01 μg/μL for Evotip loading, 3 μL of each sample was transferred to another 200 μL 96-well plate containing the calculated volume of solvent A (0.1% FA in water) by a Hamilton Microlab STAR automated liquid handling system (Hamilton, Reno, NV). Then 20 μL of each diluted sample was transferred into conditioned and equilibrated Evotip (EV-2003, Evosep, Denmark) trap column in accordance with the manufacturer’s instructions. The tips were subsequently washed with 60 μL of solvent A followed by 100 μL of solvent A for tip storage until LC/MS analysis.

### 2.3 LC–MS Analysis

#### 2.3.1 Liquid Chromatography

Plasma/serum peptides loaded onto Evotip trap columns were separated in 21 min (the 60 samples per day or SPD, a preprogrammed proprietary method) on an 8 cm × 150 μm reversed-phase column packed with 1.5 μm C18-beads (EV-1109, Evosep, Denmark) using an Evosep One LC system (EV-1000, Evosep, Denmark). The mobile phases were comprised of 0.1% FA as solvent A and 0.1% FA in ACN as solvent B, and the peptides were eluted off column at 1 μL/min flow rate within 35% solvent B.

#### 2.3.2 Data-Dependent Acquisition (DDA)

An Orbitrap Exploris 240 mass spectrometer was used to detect the LC effluents (OE240, Thermo Fisher). The spray voltage was 1850 V in the positive ion mode, and the temperature of the heated capillary was set to 280 °C. Mass spectra were acquired from 375 to 1200 *m*/*z* at a mass resolution of 60k (at 200 *m*/*z*), followed by data-dependent MS/MS with a mass resolution of 15k (at 200 *m*/*z*) for the 20 most abundant ions. The OE240 was also configured with the following settings: full MS AGC target of 3e6, MS/MS AGC target of 1e5, dynamic exclusion of 25 s, mass isolation window of 1.6 *m*/*z*, minimum intensity threshold of 1e5, and normalized HCD collision energy of 30.

#### 2.3.3 Data-Independent Acquisition (DIA)

For constructing the peptide spectral library, the OE240 MS method was set up to perform gas phase fractionation (GPF) with three scan ranges (400–560 *m*/*z*, 550–780 *m*/*z*, and 770–1000 *m*/*z*).^34^ Each acquisition consisted of a survey scan with a maximum injection time of 55 ms and an AGC target of 3e6 at a mass resolution of 60k. The MS/MS scan was acquired with 30k orbitrap resolution every 6 *m*/*z* precursor ion isolation window, AGC target 1e5, and auto for injection time. For analysis of individual samples, the mass range 400–1000 *m*/*z* was divided into 26 windows (23 ± 0.5 *m*/*z* per each isolation window) for MS/MS scans with the same parameters as the spectral library setting.

### 2.4 MS-Data Processing

#### 2.4.1 DDA Data Search

FragPipe (Ver. 17.1, https://fragpipe.nesvilab.org/) proteomics pipeline tool with MSFragger and IonQuant was utilized to search against the database (Uniprot Human, 08/06/2021, 40,870 entries including 20,435 decoys).^35^ Precursor mass tolerance for peak matching in MSFragger (Ver. 3.5) was set to ±20 ppm, and the minimum peptide length was set to 7. To increase identifications, MSBooster was selected in the PSM validation stage. IonQuant was used for MS1 quantification with the match between runs (MBR, 10 ppm *m*/*z* tolerance and 0.7 min RT tolerance).

#### 2.4.2 DIA Data Search

Thirty-six GPF-DIA measurements were performed on immunodepleted or nondepleted whole plasma samples (6 high-pH fractionations per each). To create the spectral library, GPF-DIA spectra were searched against the Uniprot human database using the MSFragger in FragPipe and the EasyPQP (Ver. 0.1.30).^36^ Individual sample DIA data were analyzed using DIA-NN (Ver. 1.8)^37^ in the “robust LC (high precision)” mode with RT-dependent cross-run normalization; MS1 and MS2 mass accuracies were set to 20 and 15 ppm, respectively, with a scan window size of 6, MBR enabled, and the curated in-house spectral library.

### 2.5 Statistics and Bioinformatics analysis

Data from DDA or DIA were processed in Perseus (Ver. 1.6.14.0, https://maxquant.net/perseus/).^38^ Proteins were considered identifiable if they contained at least one unique peptide and valid quantification values. After intensities were log2 transformed, proteins were filtered as quantifiable if the completeness of valid value is 100% across all samples. Significantly changed proteins between groups were defined by a two-sample *t* test (permutation-based FDR < 0.05, *S*^0^ = 0.01) and functionally enriched for biological processes and Reactome pathways using STRING tool (Ver. 11.5, http://string-db.org/).^39^ The property of peptide hydrophobicity was calculated using the Prot-pi peptide tool (https://www.protpi.ch/Calculator/PeptideTool) with uniquely identified peptide sequences.

### 2.6 Data Availability

Raw mass spectrometry data and database search results were deposited to ProteomeXchange with the identifier PXD038669.

## 3 Results

### 3.1 Enhancement of Identification and Quantification Using Library-Based DIA Approach

**Figure 2:**
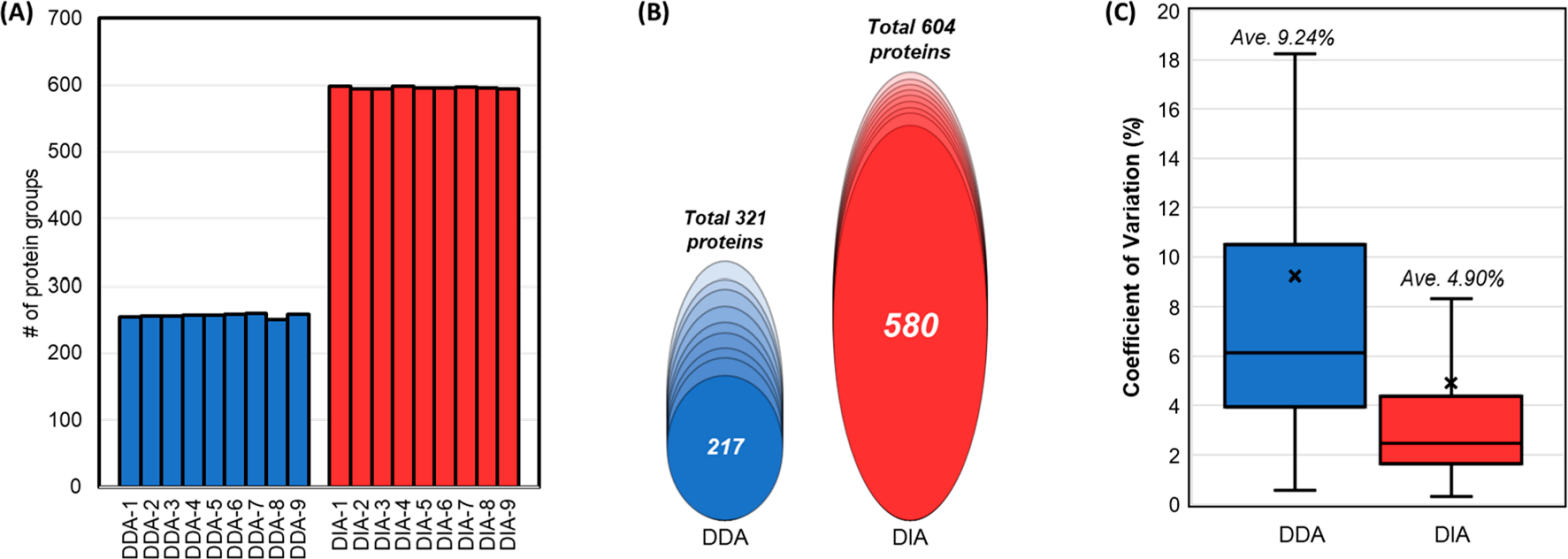
Identification and quantification comparison of DDA and DIA data. For each data acquisition, 200 ng of human plasma peptides was loaded onto the Evotip trap column without prior peptide cleanup. (A) Number of protein groups identified in nine replicates acquired in DDA or DIA mode. (B) Quantified proteins with 100% completeness in quantitative values across nine replicates in each MS mode. (C) Coefficient of variation (CV%) of quantified proteins in each MS mode.

We first assessed the effect of the data acquisition mode on the comprehensiveness of protein identification and reproducibility of quantification in plasma samples. Nine BioIVT plasma samples were prepared in microcentrifuge tubes, and LC–MS/MS data were acquired with either DDA or DIA mode. The number of protein identifications in the DIA data set reached an average of 596 protein groups (Figure 2A) using the DIA-NN software with an in-house peptide spectral library (14,121 precursors corresponding to 960 proteins), which was generated using the FragPipe workflow with high pH fractionated samples and GPF acquisitions (refer to the Methods), whereas the DDA data identified an average of 256 plasma protein groups across the replicates. In addition, the DIA MS method was superior in completeness with quantitative values among the samples: 580 protein groups were determined with 100% completeness among the 604 proteins identified in the DIA data set (Figure 2B); in contrast, 217 protein groups had all nine valid values in the DDA data set. Moreover, the mean coefficient of variation (CV) of proteins identified in the DIA data set was only about half of that in the DDA data set (4.90% vs 9.24%) despite having more than twice the number of identifications (Figure 2C). Of the 580 commonly identified proteins in the DIA data set, 89.1% (517 proteins) were detected with CV < 10%, which was significantly higher than the 72.3% (157 proteins) in the DDA data set. The higher number and less missing values in protein identifications and higher reproducibility in quantitation prove that DIA is a superior method than DDA in plasma proteomics using our current LC–MS set-ups.

### 3.2 Improved Robustness by Eliminating the Sample Desalting Procedure

**Figure 3:**
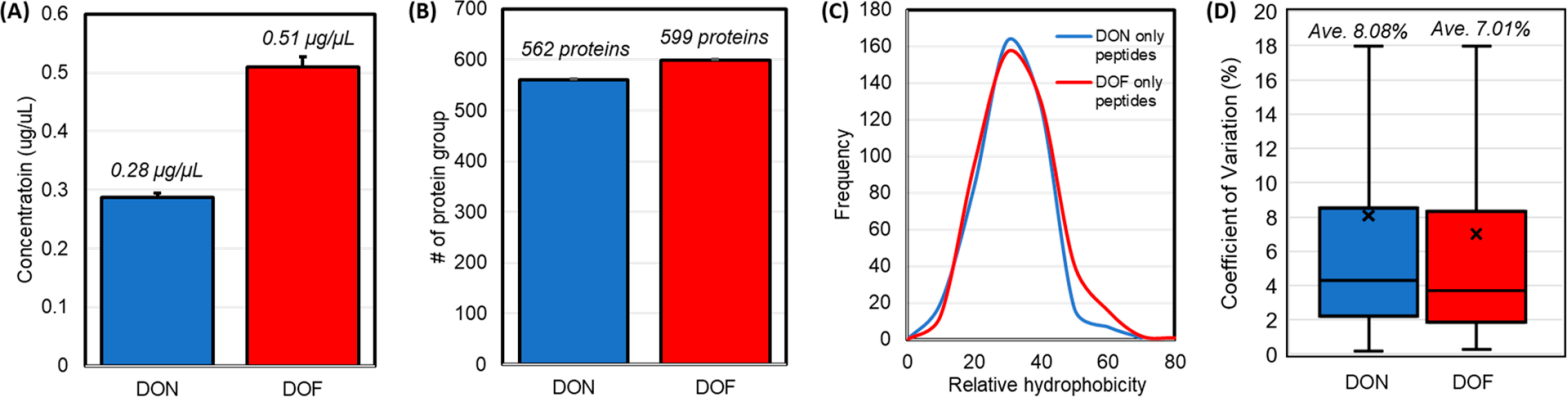
Desalting-off (DOF) vs desalting-on (DON). In DOF, 200 ng protein digest were loaded directly onto Evotips, while peptide cleanup was performed in DON using C18 solid phase extraction column before loading 200 ng desalted peptides onto Evotips. (A) Sample loss after desalting. Concentration of peptides determined using the BCA assay (*n* = 4). (B) Protein groups identified. (C) Hydrophobicity score of the uniquely identified peptide sequences in each case (418 uniquely identified peptides in DON, 455 uniquely identified peptides in DOF). Hydrophobicity of peptides was determined using the Prot-pi peptide tool. (D) CVs% of quantified proteins with 100% completeness in quantitative values were calculated across all replicates.

After proteolytic digestion, the resultant peptide samples typically need to be desalted, which not only takes time (typically ∼3 h including desalting and drying) but also results in significant sample loss (recovery typically is 50% to 60%). Since the disposable Evotip trap column for holding the samples prior to LC–MS analysis is C18 based and the loading of samples to the trap columns involves a washing step for salt removal, we assessed whether removing the desalting step immediately after protein digestion can adversely impact the performance of our proteomics workflow. To this end, plasma samples were digested in microcentrifuge tubes, and the peptide concentrations were determined using BCA colorimetric assay with (DON) or without (DOF) desalting on the regular C18 solid-phase extraction columns. Compared to the peptide concentration in the DOF sample, only 54.9% of peptides were recovered after desalting (Figure 3A). Based on the concentration measurements, 200 ng of peptides were loaded onto the disposable trap column (Evotip) and LC–MS analysis with duplicate injections per sample was performed. An average of 599 proteins (corresponding to 5625 peptides) were identified in the DOF samples, while an average of 562 proteins (corresponding to 5588 peptides) in the DON (Figure 3B). Interestingly, we discovered that the peptides only identified in DOF are more hydrophobic than those in DON (Figure 3C), implying that desalting could result in hydrophobic peptide loss during the elution step in addition to reducing total peptide yield. Similarly, for each data set, we calculated the CV for the proteins that were identified and quantified; the average CVs in DON and DOF were comparable with no statistically significant difference between them (8.08% and 7.01%, respectively, *p* = 0.104) (Figure 3D), showing that it is beneficial to eliminate the peptide desalting procedure by loading the digested peptide samples directly onto the disposable Evotips, instead of performing an additional step of peptide desalting.

### 3.3 Optimized Loading on the Disposable Trap Columns for Precise Quantification

**Figure 4:**
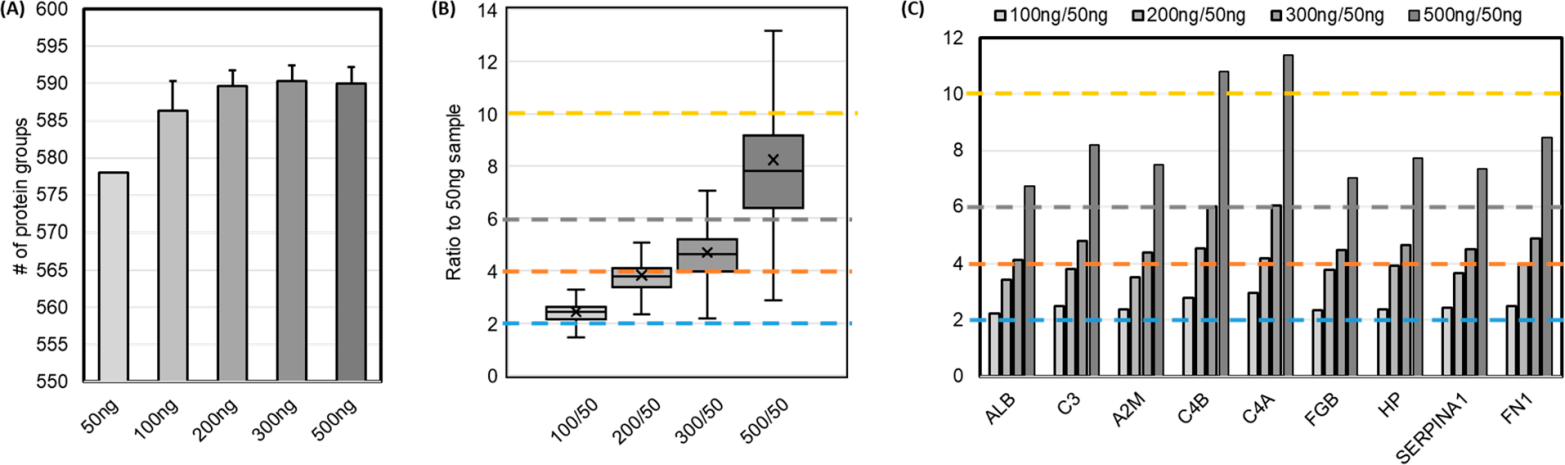
(A) Protein group identification with respect to different amounts of peptide loading, from 50 to 500 ng (*n* = 3), onto Evotip trap columns. (B) Distribution of ratio values to protein abundances of 50 ng. Each colored dot line represents the expected ratio. (C) Ratio measurement of nine FDA-approved biomarker proteins at various loadings.

For blood-based clinical proteomics work, although most of the time the amount of sample is not limited for analysis, the capacity of the disposable trap column (Evotip) does pose a loading limit and may affect the sensitivity and precision of the measurement. Three replicated plasma peptide samples were prepared and loaded onto Evotips with loading ranging from 50 to 500 ng. The total number of identifications plateaued at 200 ng peptide loading with 587 identified proteins (Figure 4A). The linearity of protein quantifications was assessed for 539 quantified proteins by calculating the intensity ratios between different loading amounts to the 50 ng sample (Figure 4B). Peptide loadings of 100 or 200 ng showed good correlation with the expected ratios (2.41 ± 0.32 and 3.78 ± 0.52, respectively), whereas higher loadings showed ratio compression (4.64 ± 0.78 and 7.81 ± 1.76 for 300 ng/50 ng and 500 ng/50 ng, respectively) and therefore underestimated the quantity of proteins in the sample. Furthermore, nine FDA-approved biomarker proteins were selected for relative quantitation, best agreement with the expected ratios was achieved at 200 ng peptide loading (Figure 4C).

### 3.4 96-Well Plasma/Serum Sample Preparation Platform for High Throughput and Reproducibility

**Figure 5:**
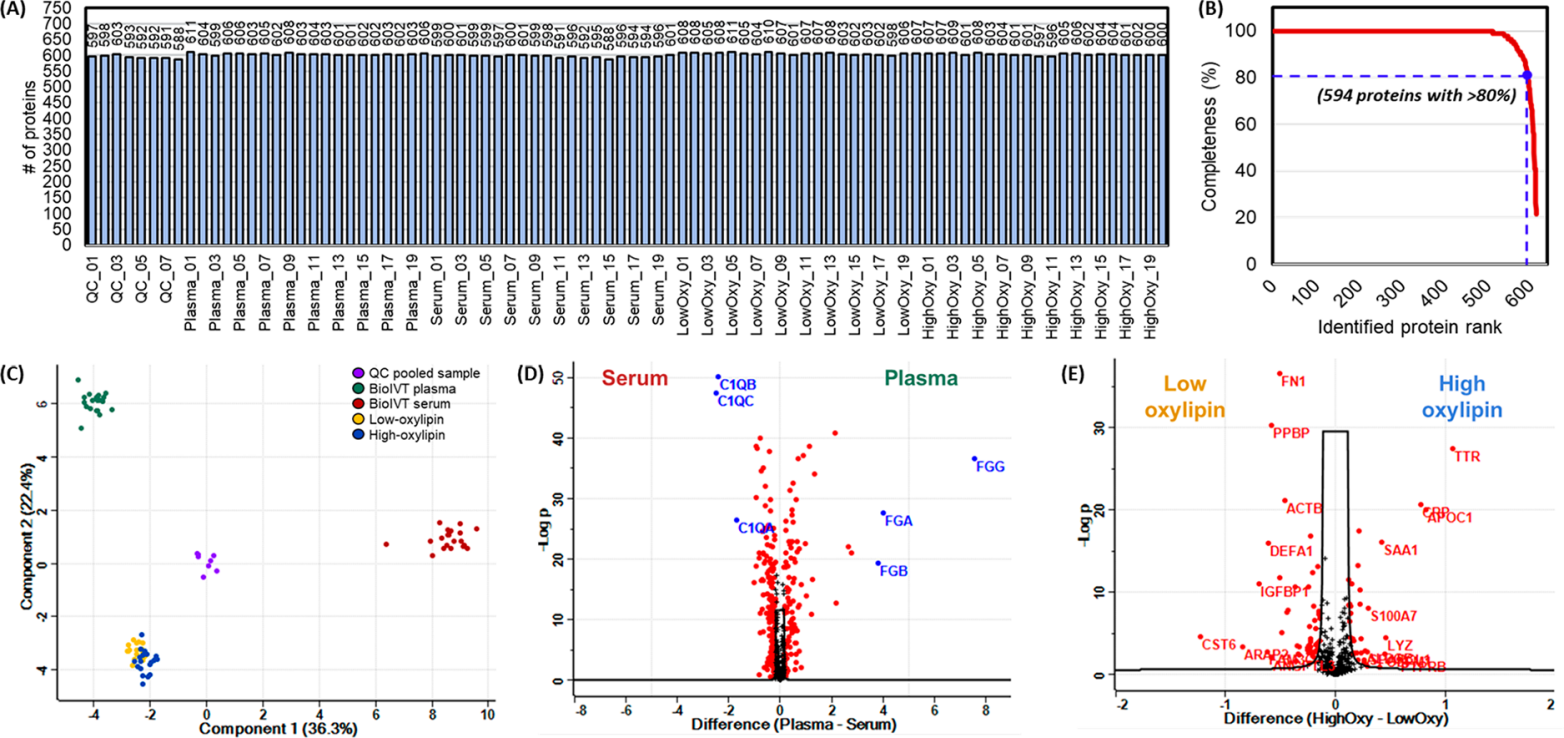
Performance evaluation of the 96-well plate sample preparation workflow. (A) Number of protein groups identified in 88 plasma/serum samples, including QCs, BioIVT plasma/serum, and low/high oxylipins plasma. (B) Completeness of quantitative data across the entire experiment, plotted against a total of 615 identified protein groups: 594 proteins with 80% and 512 proteins with 100% data completeness. (C) PCA scores plot of the 512 quantified proteins. Data were log2 transformed and colored according to sample type. (D) Comparison of pooled plasma and serum proteomes, with significantly changed proteins highlighted in red (Unpaired *t* test, permutation-based FDR < 0.05, S0 = 0.01). (E) Comparison of plasma proteomes between low and high oxylipin levels, with significantly changed proteins in red (unpaired *t* test, permutation-based FDR < 0.05, *S*_0_ = 0.01).

We next evaluated a 96-well plate-based streamlined workflow for sample preparation (see the Methods), using four different sets of samples, including pooled plasma from previously studied subjects with low or high oxylipin levels, commercial plasma or serum from BioIVT, and quality control (QC) samples prepared from pooling 10 μL aliquots from each sample in the other three sets. We identified 615 protein groups in total from the 88 plasma/serum samples, with an average of 601 protein groups (Figure 5A), 594 proteins identified having greater than 80% data completeness (Figure 5B), and 512 proteins with 100% data completeness (Supplementary Table S1). Of note, we identified 69 protein groups corresponding to 57 proteins in the FDA approved list of 109 protein biomarkers.^3^

To assess the quantitative precision, principal component analysis was used to visualize the proteomic data (Figure 5C). The four different sample sets were clearly distinguishable in PC1 and PC2, and the QC samples were grouped in the center of the PCA plot, demonstrating the high reproducibility of the high-throughput sample preparation approach and the entire workflow. Because anticoagulants were added to prevent clotting in plasma while serum was collected after blood clotting, fibrinogen alpha, beta, and gamma (FGA, FGB, and FGG) levels were upregulated in plasma,^40^ as highlighted in Figure 5D. Complement component 1 subunits (C1QA, C1QB, and C1QC) were significantly elevated in serum (Figure 5D), most likely as a result of either adding EDTA in plasma, which inhibits complement activation in blood samples or serum complement activation via the classical pathway *in vitro* during freeze/thaw cycles.^41^ We then examined whether the proteomic differences in low and high oxylipin levels, which reflects low and high intensity exercise induced stresses and inflammations,^32,33^ can be reproducibly detected (Figure 5E). The biological processes enriched in upregulated proteins in the high-oxylipin plasma are primarily related to immune response (FDR = 2.39e–08) and leukocyte mediated immunity (FDR = 9.74e–07), and the Reactome pathways annotated these proteins as pathways for innate immune system (FDR = 1.10e–09) or neutrophil degranulation (FDR = 0.00032). Therefore, we confirmed that our sHTPP platform can detect biological variations across sample sets with confidence, acceptable technical variability, and high throughput.

### 3.5 Application of sHTPP to a Large-Scale Clinical Proteomics Study

**Figure 6:**
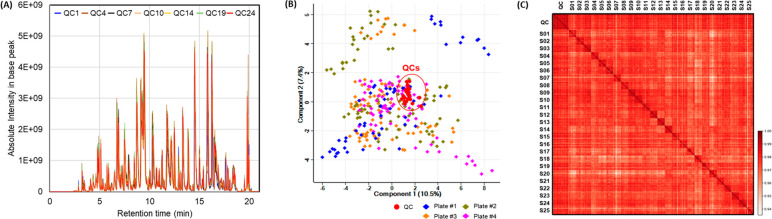
Performance characteristics of sHTPP in a clinical proteomics study. (A) Overlapped base-peak chromatograms of QC samples. QC was subjected to LC–MS analysis after every 12 individual samples. (B) PCA scores plot of the study samples, including 300 individual plasma and 25 QC samples. Plasma preparation plates were color coded. (C) Pearson correlation of samples with quantified proteins at 100% completeness.

Finally, to confirm the sHTPP platform’s reliability and robustness between different 96-well plates, we applied it to a clinical study composed of 300 plasma samples longitudinally collected from 25 subjects. Four plates were used and a total of 595 protein groups were identified, and 518 proteins were quantified with 98% completeness (Supplementary Table S2). Pooled plasma was used as QC and injected after every 12 individual samples. The overlapped chromatograms of QC samples showed very high reproducibility, and peptide desalting off did not affect the long-term performance of the LC–MS system (Figure 6A). Moreover, the QCs were consistently displayed in the center of the PCA scores plot, while individual samples in different plates were randomly distributed (Figure 6B). Because the levels of the majority of plasma proteins are specific to each individual,^14^ the longitudinal samples collected from the same individual showed higher correlation within than between individuals as shown in the correlation plot constructed from the 595 proteins quantified from all samples in this study (Figure 6C). Therefore, we validated that this platform can be used in large-scale clinical proteomics studies to reliably analyze biological variations in blood plasma samples for discovery of biomarkers and generation of hypotheses for further clinical investigation.

## 4 Discussion

In this study, we have developed an sHTPP platform that enables efficient and reproducible analysis of proteins in nondepleted blood plasma/serum samples. By using disposable trap columns for sample loading in LC–MS analysis, we eliminated the peptide desalting/purification step commonly used in proteomics sample preparation; thereby sample processing time was reduced and the hydrophobic peptide loss, which occurs during desalting and the following drying and reconstitution steps, was minimized. In addition, using an in-house generated peptide library and FragPipe and DIA-NN software, we demonstrated that DIA outperformed DDA with more than twice the sensitivity improvement and reproducibility. Through comparison of peptide loading amount on the trap columns and its effect on the quantification precision, we optimized the loading to be 200 ng for best achieved sensitivity and reproducibility.^42^ The streamlined sample preparation workflow developed based on 96-well plates showed consistent identification and quantification within plate, and its application to a 300-sample clinical study proved the high sample throughput with minimal interplate variability, as evidenced by the high reproducibility of QC samples and higher correlation between the samples within the same individual than between different individuals.

We should note that, for large-scale experiments, the number of proteins that can be consistently quantified perhaps is more important than the total number of identified proteins because only reliable detection allows for confident comparison between samples and is thus appropriate for creation of clinical assays. We benchmarked our sHTPP workflow to the previous high-throughput plasma/serum proteomics studies (Table 1). More than 600 proteins were identified with LC gradients of 60–90 min in two recent DIA-based proteomics studies, but only about half of the proteins used for quantitative analysis were with 100% data completeness.^15,18^ For more robust high-throughput analysis, the LC gradients were optimized with higher flow rate and shorter separation time of less than 20 min, but the identification and quantification were proportionally reduced regardless of whether DDA or DIA methods were used.^13,16,17,19^ Even in a study with a similarly simplified peptide purification using disposable trap columns, the completeness of quantitative values is still an issue.^20^ While consistent sample preparation plays a role, we suspect data analysis workflows also contribute to the missing data in the other studies.

**Table 1:** Benchmarking sHTPP with Previous High-Throughput Plasma/Serum Proteomics Studies (NS: Not specified)

	Laboratory setting						Results	
	LC	Mass Spec	Peptide Purification	Retention Time	MS mode	Data Processing Tool	Protein ID	Quantified Protein (Completeness, %)
Bennike et al.^15^	*Eksigent*	*Q-Exactive*	Yes	60 min	DIA	Spectronaut	664	241 (100)
Han et al.^18^	*Ultimate 3000*	*Q-Exactive plus*	Yes	90 min	DIA	Spectronaut	608	484 (50)
Johansson et al.^17^	*Evosep*	*timsTOF pro*	Yes	21 min	DIA-PASEF	Spectronaut	393	NS
Messner et al.^13^	*Waters H-Class*	*Triple TOF 6600*	Yes	5 min	DIA	DIA-NN	311	182 (100)
Mc Ardle et al.^19^	*Evosep*	*OE480*	Yes	21 min	DIA	OpenSWATH	471	117 (60)
Geyer et al.^16^	*EASY-nLC 1000*	*Q-Exactive HF*	NS	20 min	DDA	MaxQuant	313	284 (100)
Mi et al.^20^	*Evosep*	*timsTOF pro*	No	11.5 min	DDA-PASEF	FragPipe	269	170 (60)
Woo et al. [this study]	*Evosep*	*OE240*	No	21 min	DIA	FragPipe/DIA-NN	615	512 (100)

To our knowledge, the sHTPP platform developed in the current study excels in throughput, number, and completeness of identified proteins, allowing for comprehensive analysis of plasma proteins in clinical samples with high quantitative reproducibility. For implementation of the sHTPP workflow, in this study, we used general laboratory equipment (e.g., multichannel pipettes, a microplate vortexing incubator, etc.) in sample preparation except for a liquid handler to dilute the peptides in the 96-well plate to the same concentration for Evotip loading. This suggests that the sHTPP workflow can be extended to most mass spectrometry laboratories for higher accessibility. This sample preparation workflow could also be the foundation for a hands-free automated sample preparation protocol using a liquid handling robot. In conclusion, the sHTPP workflow enables high-throughput, robust proteomic measurements of blood samples in large scale clinical studies, has potential in clinical diagnosis, therapeutic evaluation, and may facilitate hypothesis generation in mechanistic investigations of diseases.

## Supporting Information Available

(Table S1) List of 512 quantified protein groups with log-transformed intensities. Samples include 20 BioIVT plasma, serum, and low/high oxylipin level plasma, as well as 8 pooled QC plasma (XLSX)
(Table S2) List of 595 identified plasma proteomes with log-transformed quantities. Samples include 300 individual plasma and 25 pooled QC plasma (XLSX)
